# ALGA-DenseNet ground-based cloud classification network based on multi-scale features

**DOI:** 10.1371/journal.pone.0333999

**Published:** 2025-10-30

**Authors:** Binbin Tu, Haoyuan Zhou, Xiaowei Han, Jiawei Bao, Linfei Zhao, Nanmu Hui

**Affiliations:** 1 College of Intelligent Science and Information Engineering, Shenyang University, Shenyang, Liaoning, China; 2 College of Science, Shenyang University, Shenyang, Liaoning, China; Pacific Northwest National Laboratory, UNITED STATES OF AMERICA

## Abstract

Automatic recognition of ground-based clouds is crucial for meteorology and especially for the operational safety of Unmanned Aerial Vehicles (UAVs), but it is challenged by variable cloud shapes, complex lighting, and background interference. This paper introduces ALGA-DenseNet, an improved DenseNet model with a multi-scale attention mechanism. The model employs Color Jitter to enhance image robustness and improve learning of intra-class variations and inter-class differences. It incorporates Adaptive Local and Global Attention (ALGA) to merge features, enhancing feature selection. Additionally, it integrates mixed and depthwise separable convolutions to optimize multi-scale feature extraction, reducing parameters and computational complexity. Furthermore, integrating a Vision Transformer (ViT) and Dynamic Multi-head Attention (DMA) enhances representation of complex cloud features. Experimental results show recognition accuracies of 97.94% on the TJNU (Tianjin Normal University) Ground-based Cloud Dataset (GCD) and 97.25% on the Cirrus Cumulus Stratus Nimbus (CCSN) dataset. This indicates the model’s capability for fine-grained, multi-scale extraction of cloud textures, shapes, and color features, along with strong generalization performance.

## 1 Introduction

Clouds, composed of vast numbers of water droplets or ice particles suspended in the atmosphere, cover more than 50% of Earth’s surface [1]. As a prevalent low-altitude weather phenomenon, ground-based clouds pose a significant challenge to the perception capabilities and flight safety of Unmanned Aerial Vehicle (UAV) visual systems. More broadly, clouds play a critical role in regulating Earth’s radiation balance and influencing weather and climate patterns [2]. Nowadays, precise cloud observation techniques are essential for various practical applications such as optical remote sensing [3], weather forecasting [4], precipitation estimation [5], ensuring the safety of low-altitude UAV operations, and deep-space climate observation missions [6].

Specifically, the ability to automatically identify cloud types is crucial for proactive risk assessment in UAV missions. For instance, recognizing Cumulonimbus clouds can preemptively warn of severe turbulence, icing, and thunderstorms, which pose a direct threat to the aircraft. Similarly, identifying low-lying Stratus or Nimbostratus clouds indicates poor visibility and potential widespread icing conditions that could compromise optical sensors and flight stability. This automated recognition enables intelligent decision-making at two critical stages: for pre-flight checks, determining if conditions are safe for takeoff, and for in-flight operations, allowing for dynamic path planning to avoid developing weather hazards. Cloud formation results from the interplay of atmospheric motions, airborne impurities, water vapor, and conditions such as temperature and sunlight [7]. Clouds continuously affect atmospheric radiation, temperature, and humidity, playing a central role in Earth’s water cycle, radiation balance, and climate change [8–11]. These interactions introduce significant uncertainties to global climate and local weather phenomena. Therefore, the variability, causes, and impacts of clouds are highly unpredictable, making cloud observation a crucial and challenging task. To address this challenge, researchers have employed various remote sensing technologies. For example, Valk et al. investigated the automated detection and classification of Cumulonimbus (CB) and Towering Cumulus (TCU) cloud morphologies using radar and satellite data [12]. Similarly, Henken et al. studied the automatic detection of Cb/TCu clouds using MSG-SEVIRI satellite cloud physical properties and weather radar observations [13]. With advancements in remote sensing and computer vision technologies, research into automated ground-based cloud recognition systems has become a focus.

In recent years, the rise of deep learning technologies, particularly convolutional neural networks (CNNs), has revolutionized image recognition. Deep learning models automatically extract features from image data and classify them without manually designed features, significantly enhancing recognition accuracy and efficiency. In ground-based cloud recognition, deep learning methods enable the automatic analysis and classification of extensive cloud imagery data [14–16].

Cloud height, cloud cover, and cloud type are the three main aspects of cloud observation and have been extensively studied [16–20]. For ground-based cloud classification by cloud type, extracted features include color, texture, and structure. Researchers have developed numerous methods utilizing these features for classification tasks [21–23]. However, cloud shapes are susceptible to weather factors, making their forms highly variable [24]. Variability in cloud forms, differences in lighting conditions, and background interference decrease classification accuracy and complicate feature extraction. Currently, research into ground-based cloud image classification remains exploratory.

As deep learning models evolve, researchers increasingly focus on comprehensive and in-depth feature extraction from cloud images. Heinle et al. [25] tested various features by extracting cloud image characteristics—including spectral, co-occurrence matrix, and cloud cover features—achieving an accuracy of approximately 97% across all categories, notably in dense cloud types like cumulonimbus. Liu et al. [26] developed an infrared image structural feature method for cloud classification, extracting metrics such as cloud grayscale mean, coverage, edge sharpness, and the distribution of clouds and gaps, effectively distinguishing cirrus, cumulus, and stratus clouds with an accuracy of 90.97%. Kazantzidis et al. [21] employed a multicolor criterion to better classify fragmented or hazy cloud images, achieving an average accuracy of about 87%. Wacker et al. [27] used long-wave radiation as supplementary information for cloud classification, enhancing accuracy by nearly 10% compared to using image-based data alone, with an average accuracy reaching 90%. Dev et al. [28] applied a classification method based on texture tokens, combining color and texture information to achieve an average accuracy of nearly 95% on the Singapore Whole sky IMaging CATegorization (SWIMCAT) dataset. Li et al. [29] treated whole-sky images as collections of microstructures, extracting and clustering them to build a feature dictionary and encode the images. This method, effectively captures texture features, achieved a classification accuracy of 90% for five types of cloud images. Zhi et al. [30] proposed a Dense Scale-Invariant Feature Transform (Dense_SIFT) algorithm to extract texture features from cloud images. This scale-invariant approach densely samples feature points across the image, ensuring stability and better describing cloud textures. Li [31] used image enhancement techniques such as Gamma Correction to optimize image brightness and contrast, clearly delineating cloud textures and structural features, effectively identifying the morphology of different cloud layers, cloud gaps, edge sharpness, and the arrangement of cloud masses.

To better extract texture, shape, and color features from images, integrating multi-scale feature attention mechanisms is a current focus in image recognition research [32]. Ju et al. [33] introduced an Adaptive Feature Fusion and Attention Mechanism (AFFAM) to enhance multi-scale target detection performance. AFFAM adjusts feature maps of varying scales via path layers and sub-pixel convolution layers to ensure effective integration of multi-scale features. AFFAM also employs global and spatial positional attention mechanisms to adaptively learn channel correlations and spatial feature importance. This approach allows the network to focus on key features across multiple scales, significantly improving detection accuracy and robustness, especially in complex scenarios. Qing et al. [34] proposed a Multi-scale Residual Convolutional Neural Network (MRA-NET) incorporates an efficient channel attention network. This network uses multi-scale residual structures on dimensionally reduced image data to extract spatial and spectral features, addressing vanishing gradients in deep network training. The improved attention mechanism reduces network parameters and computational load via local cross-channel interaction and one-dimensional convolution, effectively allocating weights to features across different channels. By utilizing convolutional kernels of various scales and sizes, the network captures image features at different levels, from fine to coarse granularity. Emin et al. [35] developed a hyperspectral image classification method based on Squeeze-and-Excitation Networks (SENet), depthwise separable convolutions, and multi-branch feature fusion, enriching feature extraction while minimizing total parameters and reducing the number of trainable parameters to enhance classification performance.

In summary, this paper presents ALGA-DenseNet, a ground-based cloud classification network based on multi-scale features, with the following main contributions:

(1) Applying Color Jitter to mitigate illumination variance, enhancing the model’s focus on robust morphological features over superficial color cues.
(2) It utilizes the ALGA mechanism, integrating local and global features via element-wise multiplication within convolutional modules, combined with dynamic multi-head attention mechanisms and linear classification layers for precise cloud categorization.
(3) The network integrates mixed convolutions, depthwise separable convolutions, and ViT capabilities for long-range dependency modeling to aggregate global and local features, enhancing the capture of multi-scale key features.
(4) Extensive experiments on the large-scale and challenging TJNU Ground-based Cloud Dataset (GCD) demonstrate that our proposed ALGA-DenseNet achieves state-of-the-art performance, surpassing existing methods in classification accuracy and robustness.

## 2 Data

### 2.1 GCD ground-based cloud dataset

This study employs the TJNU Ground-based Cloud Dataset (GCD) [36], which contains 19,000 images collected from nine provinces in China captured from a ground-based platform. All images were professionally annotated by meteorologists. The dataset’s classification scheme is based on the World Meteorological Organization (WMO) standards, adapted for machine vision by grouping clouds with high visual similarity. This results in seven distinct sky condition categories: (1) Altocumulus and Cirrocumulus; (2) Cumulonimbus and Nimbostratus; (3) Clear Sky; (4) Cirrus and Cirrostratus; (5) Cumulus; (6) Stratocumulus, Stratus, and Altostratus; and (7) Mixed Cloud. The ‘Clear Sky’ class is defined as images with cloud cover of 10% or less. The dataset is officially split into 10,000 training images and 9,000 test images. Table 1 provides details for each category.

**Table 1:** GCD Dataset Information.

Type	Description	Number
Altocumulus and Cirrocumulus	Patchy layers or sheets of small cloud elements appearing in ripples	1475
Cumulonimbus and Nimbostratus	Dense, dark, precipitation-producing clouds of great vertical extent	5764
Clear Sky	An unobstructed sky with a total cloud cover of ten percent or less	3739
Cirrus and Cirrostratus	Thin wispy ice-crystal clouds appearing as filaments or a transparent veil	1906
Cumulus	Mid-level or low-level clouds with flat bases	1525
Stratocumulus, Stratus, and Altostratus	Gray or white sheet-like clouds that may form layers, patches, or rolls	3636
Mixed Cloud	Two or more distinct cloud types present without a single dominant form	955

### 2.2 Data processing

**Fig 1:**
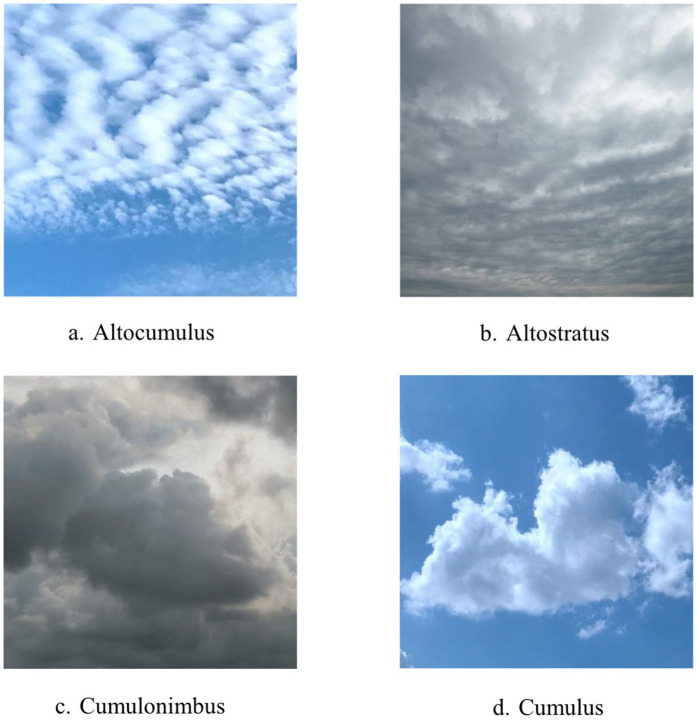
Ground-Based Cloud Samples from the Dataset.

All images are in JPEG format with a balanced distribution across categories. Examples from the dataset are shown in Fig 1.

**Fig 2:**
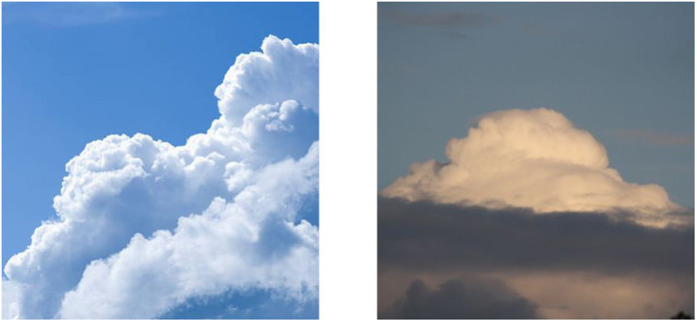
Cumulus Clouds Under Varying Lighting Conditions.

Fig 2 displays cumulus clouds under various lighting conditions, highlighting significant lighting variations and within-class differences in the dataset. Preprocessing and data augmentation techniques have been applied to enhance model generalization and reduce overfitting.

**Fig 3:**
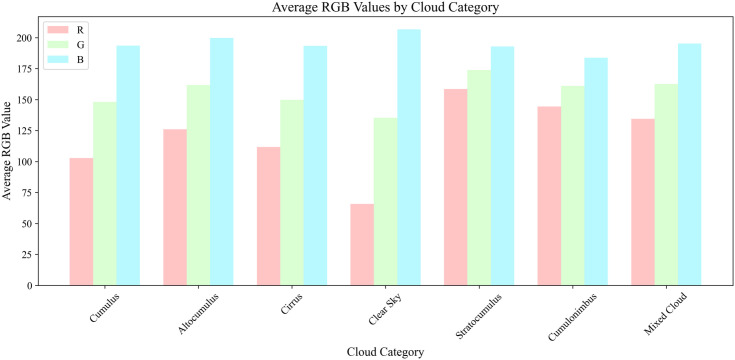
Average RGB Values by Cloud Category.

A quantitative analysis of the original dataset was performed to document the color characteristics of each cloud category. Fig 3 presents the average Red, Green, and Blue channel values for all seven classes. The results reveal systematic color variations across categories that are highly susceptible to changing illumination. This finding underscores the unreliability of color as a primary feature and motivates our use of data augmentation to enhance the model’s robustness against these illumination variations.

In ground-based cloud detection, color features are commonly used to distinguish clouds from the sky through binary image segmentation. Specifically, the difference between the red and blue channels often reflects sky irradiance, effectively differentiating clouds from the sky in images [25].

**Fig 4:**
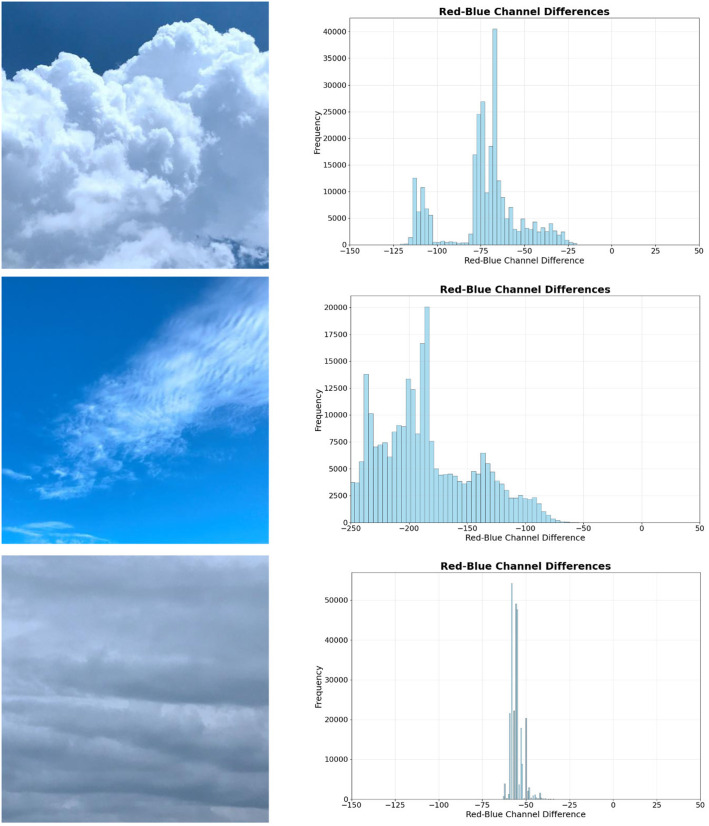
Histogram of the red-blue channel differences.

However, in cloud recognition, color features are often overlooked due to subtle color differences among cloud types. As illustrated in Fig 4, the Histogram of the red-blue channel differences reveals variations among different cloud categories. In fact, the distribution of colors and relative local color changes significantly aid in characterizing and distinguishing image features, reflecting the distinct characteristics of various cloud formations.

To ensure methodological rigor and prevent data leakage, we combined the official 10,000 training and 9,000 test images, and then re-partitioned the resulting 19,000-image dataset into training, validation, and test sets using a 70%, 20%, and 10% ratio, respectively. This division was performed prior to any data augmentation to guarantee the integrity and independence of our evaluation sets. A consistent preprocessing pipeline was then applied across all subsets. This pipeline began with resizing images to 256 × 256 pixels. Following resizing, the images were converted into PyTorch tensors, a process that inherently scales pixel values to the [0.0, 1.0] range. Subsequently, we applied channel-wise standardization using pre-calculated mean and standard deviation values to align the input data distribution with that expected by the pre-trained model backbone. To enhance model robustness and mitigate overfitting, a series of augmentation techniques—including random rotations, horizontal flipping, and color jittering—were applied exclusively and on-the-fly to the training set during the training process. This strategy provides a fair and accurate evaluation of the model’s generalization capabilities without contaminating the evaluation sets.

**Fig 5:**
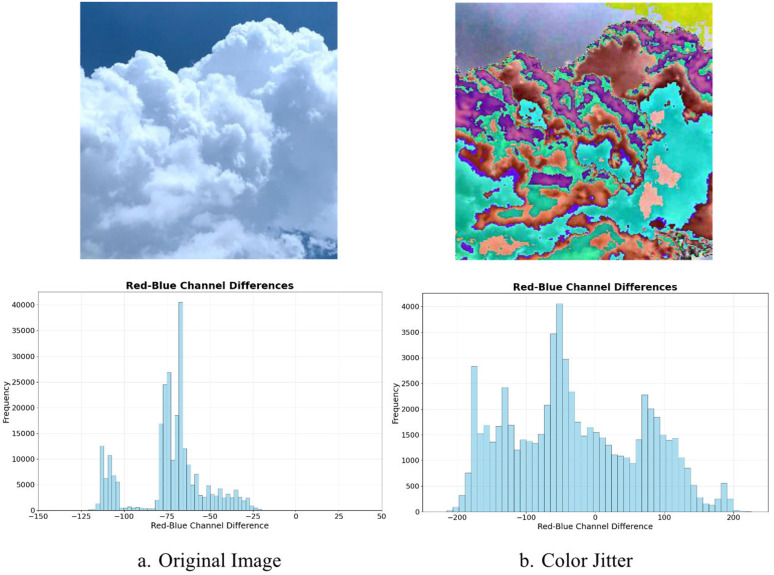
Effects of Color Jitter.

Fig 5 shows the histogram of differences between the red and blue channels. The original images exhibit distinct peaks or complex structures, indicating that the distinction between clouds and the sky relies on these differences, with red indicating strong illumination and high brightness. After Color Jitter, the histogram becomes more concentrated and smoother, with reduced peak effects, indicating reduced differences but a more uniform overall distribution. By minimizing color differences between channels and enhancing color distribution uniformity, the model more effectively extracts fundamental features of similar clouds under varying lighting conditions without interference from lighting-induced color changes. Moreover, a uniform color distribution under consistent lighting aids the model in distinguishing different cloud types. Each cloud type has unique shape and color characteristics. Color Jitter accentuates these features during training, thereby improving cloud classification accuracy and model feature extraction efficiency.

## 3 ALGA-DenseNet model architecture

**Fig 6:**
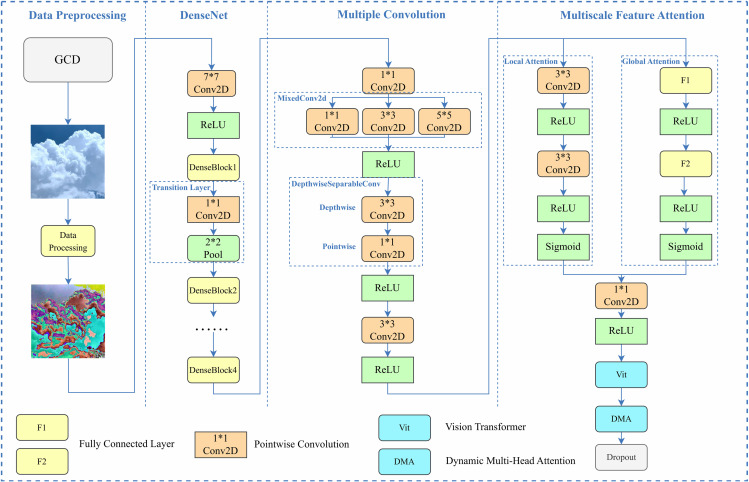
General Network Structure Diagram.

Fig 6 shows the ALGA-DenseNet model architecture developed in this paper. It includes a multi-scale feature attention module comprising ALGA, ViT, and DMA. DenseNet efficiently reuses features through its dense connectivity mechanism, enhancing feature representation richness and network training efficiency [37]. The model employs a pre-trained DenseNet121 as the feature extractor and ViT as the classifier. Building on a traditional dual-model approach, the model incorporates an ALGA module combined with ViT, enhancing feature extraction and representation capabilities. This enables efficient local feature extraction and comprehensive global structure modeling, significantly improving ground-based cloud recognition accuracy and robustness.

Preprocessed input images are fed into DenseNet121’s four DenseBlocks for feature extraction. After dimensionality reduction, the feature maps are input into mixed convolution blocks to form multi-scale feature maps. Depthwise separable convolution modules further reduce model parameters and complexity through depthwise and pointwise convolutions. The ALGA module generates attention weights through local convolution and global pooling, enhancing feature representation after merging with the input feature maps. Channel numbers are adjusted to fit ViT. After removing the classification head, the feature maps are segmented into patches, undergo linear transformation and positional encoding, and are modeled by the Transformer encoder using a self-attention mechanism. In the DMA module, multiple heads calculate attention weights and outputs, which are summed by a dynamic weight network to produce the final attention output. Output features are mapped to class numbers through a fully connected layer, and classification predictions are made using the Softmax function.

### 3.1 DenseNet module

**Fig 7:**
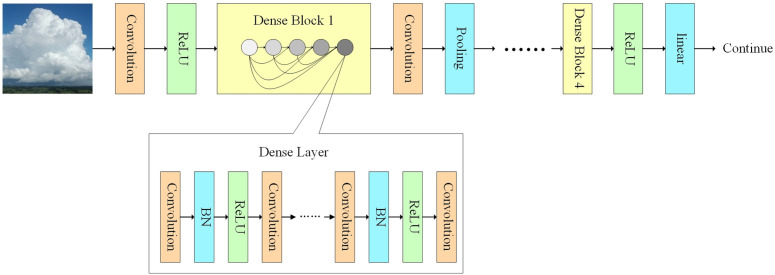
Diagram of the DenseNet Module Structure.

The DenseNet module includes four dense convolution blocks, each employing dense connectivity. The output of each convolutional layer not only serves as input for the next layer but is also concatenated with the outputs of all preceding layers along the channel dimension. Each convolutional layer receives the combined outputs of all prior convolutional layers as its input. Between two dense blocks, a transition layer typically reduces the feature maps’ dimensions and channel number through 1 × 1 convolution and 2 × 2 average pooling, preparing the input for the next dense block. The four dense blocks contain 6, 12, 24, and 16 convolution layers respectively. The detailed structure shown in Fig 7.

DenseNet’s dense blocks feature multidirectional connectivity, not just unidirectional. Each layer is connected to every preceding layer, allowing the network to utilize features more effectively while maintaining lower complexity [38]. This architecture enables DenseNet to excel in ground-based cloud image recognition tasks, particularly because it maintains strong performance even with fewer parameters.

### 3.2 Multiconvolution module

Clouds typically vary in shape and size, and using convolutional kernels of different sizes allows capturing multi-scale features [39]. Smaller kernels capture fine details, while larger ones extract broader structural information.

**Fig 8:**
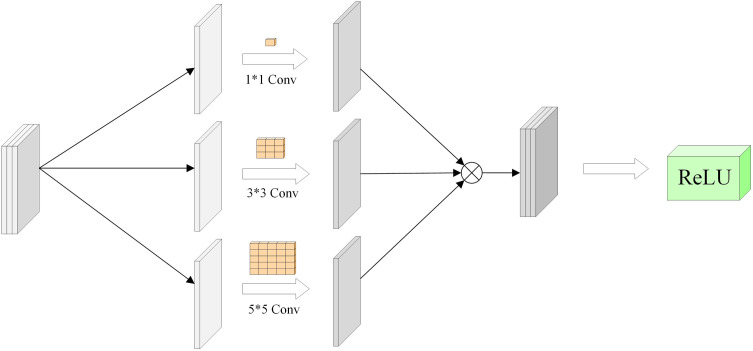
Diagram of the Mixed Convolution Module Structure.

As shown in Fig 8, mixed convolution combines three sizes of convolutional kernels to simultaneously process features of different scales, enhancing the model’s ability to recognize complex cloud formations and adapt to various cloud types [40]. Outputs from different convolutional kernels are concatenated along the channel dimension, increasing feature diversity and improving classification accuracy.

Depthwise separable convolution divides standard convolution into depthwise and pointwise convolutions: depthwise convolution independently processes each channel’s information, and pointwise convolution merges information across channels. This significantly reduces computational and parameter demands, lowers complexity, increases operational speed, and saves storage space [41].

In the standard depthwise separable convolution architecture, the features extracted by the depthwise and pointwise convolutions are typically concatenated along the channel dimension. While effective, this approach treats spatial and channel-wise features as separate, independent blocks of information.

To foster a more intricate interaction between these two feature types, we introduce a modification to this structure. Instead of concatenation, we fuse the outputs of the depthwise and pointwise convolutions using element-wise multiplication, as formulated in Equation (1).


$$\{\;\;\;\begin{array}{c} {}*20lD\left(x\right)=x*K_{d}\\ P\left(x\right)=x*K_{p}\\ O=D(x)*P(x)=(x*K_{d})*(x*K_{p}) \end{array}$$ (1)

Where *x* is the input feature map, *K*_*d*_ is the depth convolution kernel, *K*_*p*_ is the pointwise convolution kernel, * is the convolution operation, *D*(*x*) is the depth convolution, *P*(*x*) is the pointwise convolution and *O* is the traditional depthwise separable convolution.

This modification serves two critical purposes. First, it acts as a feature gating mechanism, where the channel-specific features from the pointwise convolution dynamically recalibrate the spatial features from the depthwise convolution. This allows the model to selectively emphasize more informative spatial patterns. Second, this non-linear fusion enhances the model’s expressive power, enabling it to learn more complex feature representations.

### 3.3 Multiscale feature adaptive enhancement module

Building on the multiscale attention mechanisms designed by Ju et al. [33], Qing et al. [34], and Emin et al. [35], this paper introduces the ALGA module, ViT, and DMA module to implement multiscale feature attention. By integrating various network architectures and attention mechanisms, the model aims to enhance performance in complex image processing tasks.

**Fig 9:**
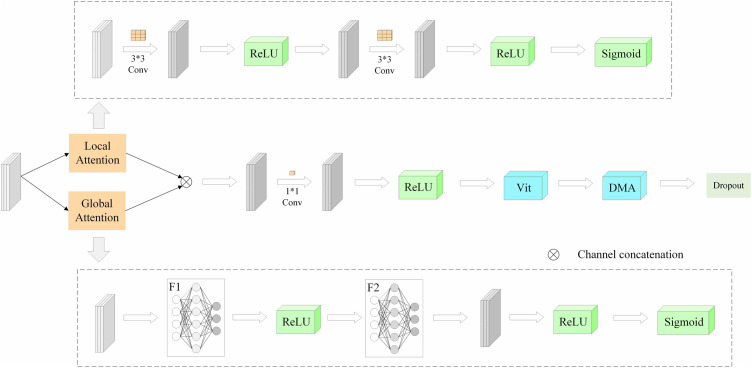
Diagram of the ALGA Module.


As shown in Fig 9, the ALGA module combines local and global features, enhancing the model’s feature extraction capabilities through adaptive adjustment of attention weights. The local and global attention mechanisms are connected via element-wise multiplication, effectively integrates local and global attention features. Local attention captures detailed information, while global attention captures contextual information, enriching and broadening the feature representation when combined. Element-wise multiplication modulates feature values at each position and channel by corresponding attention weights. Features at positions and channels with high weights are preserved or amplified, while those with low weights are suppressed, enhancing the model’s focus on important features. Element-wise multiplication is defined as follows:

$$X^{\prime }=X⊙A_{local}⊙A_{global}$$ (2)

Where ⊙ is the element-wise multiplication, *X* is the feature map, $X^{\prime }$is the adjusted feature map, *A*_*local*_ and *A*_*global*_ are dynamic attention weights that balance the contribution of local and global features, respectively.

In this paper, the ViT is used to extract global image features. ViT utilizes a self-attention mechanism to process a series of small patches from segmented images, capturing local features and understanding the global context through long-range dependencies. The pre-trained model used is vit_b_16, well-suited for complex image recognition tasks. By removing the original classification head and replacing it with a custom linear layer, ViT integrates better into the overall network architecture, adapting to specific classification tasks. Feature maps processed by the ALGA and mixed convolution structures are further refined through ViT, enhancing the model’s understanding of image details and optimizing its ability to capture image structures globally. The extraction of these global features provides rich contextual information for subsequent attention mechanisms.

To capture global contextual information, we integrate a pre-trained ViT module into our architecture following the ALGA block. Our integration strategy is distinctive: instead of appending a few Transformer layers, we utilize the entirety of a vit_b_16 model as a powerful, secondary feature extractor. To achieve this, the 256 × 224 × 224 feature map from the preceding convolutional stage is first passed through a 1 × 1 convolutional layer to reformat it into a 3 × 224 × 224 tensor. This “pseudo-image” tensor, which encapsulates rich semantic features, is then fed as a standard input to the vit_b_16 model. By leveraging the full pre-trained ViT architecture—which internally performs its own patch embedding, positional encoding, and multi-head self-attention across multiple encoder layers—our model benefits from its powerful capability to model long-range dependencies. We remove the original classification head of the ViT, using the resulting 768-dimensional feature vector as a global feature representation which is then passed to the Dynamic Multi-head Attention module for further processing.

**Fig 10:**
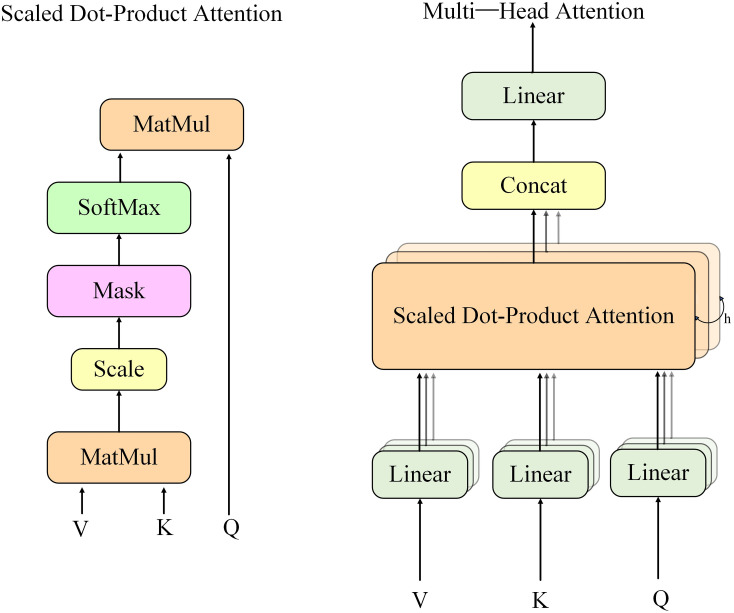
Diagram of the DMA Module.

Dynamic Multi-head Attention is a key component in this paper for fine-grained feature adjustment. Its structure is shown on the right side of Fig 10, and unlike the scaled dot-product attention on the left side, by processing features in separate heads, DMA allows the model to independently learn information in different representational subspaces, capturing complex and layered feature dependencies [42]. In this model, DMA not only enhances feature interactions but also introduces dynamics that allow attention weights to adjust in real-time based on input features. Integrated with the ALGA module, DMA strengthens the consolidation of global information while considering local details. By combining ViT and DMA, the model captures global features and dynamically adjusts weights for different features, enabling adaptive attention to critical features and enhancing the understanding and classification of complex images. In conjunction with the ALGA module, the model balances global information integration with attention to local details. This dynamic adjustment allows the model to remain efficient and accurate across various scenarios. Important features receive more attention through enhanced weights, while less important information is suppressed, enabling adaptive extraction of key information crucial for handling complex image tasks.

## 4 Experimental results and analysis

### 4.1 Experimental results

The model’s training parameters are set to ensure effective learning and adaptation to complex image recognition tasks. To mitigate overfitting, we primarily leverage transfer learning with pre-trained backbones and incorporate regularization layers such as Dropout and Batch Normalization within the model’s architecture. The Adam optimizer is selected with an initial learning rate of 0.0001. To ensure stable convergence and aid in preventing overfitting, a dynamic learning rate scheduling strategy was employed. Specifically, the learning rate was halved if the validation loss did not show improvement for five consecutive epochs, with a lower bound of 0.00001. The training batch size is 32, with validation and test batch sizes of 16 and 32, respectively. Four worker threads handle data loading, improving training efficiency and GPU utilization. The CrossEntropyLoss function is adopted, suitable for multi-class classification problems, effectively measuring the discrepancy between predicted and actual label distributions. With these parameters, the model is trained for 10 epochs, yielding training and testing results.

To maximize the benefits of pre-training and rigorously mitigate the risk of overfitting, we adopt a parameter-efficient fine-tuning approach by freezing the weights of both the DenseNet-121 and the Vision Transformer feature extractors. While the full ALGA-DenseNet architecture comprises approximately 97.6 million parameters, this freezing strategy ensures that only a small, critical subset is trained on the cloud dataset. Specifically, the number of trainable parameters is reduced to just 4.8 million, 4.93% of the total, with the remaining 92.8 million parameters remaining fixed. These trainable parameters are located exclusively within our novel ALGA and DMA fusion modules and the final classification head. This forces the model to focus on learning the optimal strategy for integrating local and global features, rather than re-learning low-level features, thereby ensuring robust generalization even with a limited training dataset.

**Fig 11:**
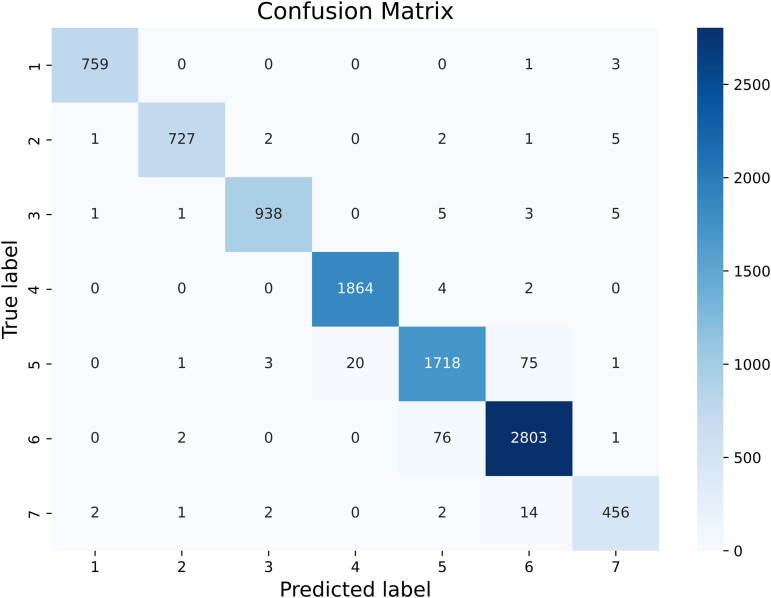
Confusion Matrix. The numerical labels correspond to the cloud categories as follows: (1) Altocumulus and Cirrocumulus; (2) Cumulonimbus and Nimbostratus; (3) Clear Sky; (4) Cirrus and Cirrostratus; (5) Cumulus; (6) Stratocumulus, Stratus, and Altostratus; and (7) Mixed Cloud.

The confusion matrix in Fig 11 reflects the recall rate for each category, i.e., the proportion of correctly predicted instances within that category. The darker the diagonal of the matrix, the better the recognition performance. Each category’s recall rate is around 96%, indicating high recognition for all categories.

Precision indicates the likelihood that a predicted positive is correct, recall reflects the probability of a true positive being predicted, and the F1 score harmonizes precision and recall. As shown in Table 2, the model demonstrates excellent accuracy, recall, and F1 scores, particularly achieving a 99% precision in recognizing altocumulus clouds.

**Table 2:** Category Results.

Category	Accuracy	Recall	F1-Score
Altocumulus and Cirrocumulus	0.9948	0.9948	0.9948
Cumulonimbus and Nimbostratus	0.9932	0.9851	0.9891
Clear Sky	0.9926	0.9843	0.9884
Cirrus and Cirrostratus	0.9894	0.9968	0.9931
Cumulus	0.9507	0.9450	0.9478
Stratocumulus, Stratus, and Altostratus	0.9669	0.9726	0.9697
Mixed Cloud	0.9682	0.9560	0.9621
Model	0.9794	0.9764	0.9779

### 4.2 Ablation studies

To validate the effectiveness of the proposed network structure, we conducted a series of ablation studies, where we systematically removed or replaced key components of the model to evaluate their individual contributions on the same ground-based cloud dataset. The details are as follows:

(1) Removing depthwise separable convolution: Classify ground-based clouds without using depthwise separable convolution, substituting standard convolution layers to assess its contributions to feature extraction and computational reduction.
(2) Removing mixed convolution: Replace mixed convolutions with single-type convolutions to verify the effectiveness of mixed convolutions in integrating multi-scale information.
(3) Removing local attention: Eliminate the local attention mechanism and rely solely on global features to explore the role of local attention in capturing detailed features.
(4) Removing global attention: Remove the global attention mechanism and rely solely on local features to analyze the importance of global attention in integrating overall information.
(5) Removing ALGA: Remove both local and global attention mechanisms to observe their impact on performance, focusing on validating the ALGA module’s contribution.
(6) Removing DMA: Eliminate the DMA module to test its role in information integration and assess its capability to dynamically distribute weights among different features.

(7) Removing ALGA and DMA simultaneously: Remove these two modules together to verify their synergistic effects and their importance in model performance.
(8) Using the complete ALGA-DenseNet model: Use the complete model as a baseline to validate the effectiveness of each module.

The results in Table 3 demonstrate that removing any module leads to a decrease in classification accuracy, particularly when ALGA and DMA are removed together, resulting in the lowest accuracy of 92.79%. This underscores the crucial role each module plays in feature extraction and integration. Comparisons of ablation study results yield the following conclusions:

**Table 3:** Ablation Study Results.

Ablation Methods	Accuracy
Removing Depthwise Separable Convolutions	94.34%
Removing Mixed Convolutions	95.13%
Removing Local Attention	93.26%
Removing Global Attention	95.13%
Removing ALGA	93.17%
Removing DMA	94.65%
Removing ALGA and DMA	92.79%
**ALGA-DenseNet**	**97.94%**

(1) Depthwise separable convolutions reduce computational load while effectively extracting crucial features, and mixed convolutions enhance feature extraction by integrating information across multiple scales. Removing these modules significantly impairs model performance, indicating their key roles in balancing efficiency and effectiveness.
(2) Local attention mechanisms capture detailed features, while global attention helps integrate overall information. Upon removing local and global attention, accuracy falls to 93.26% and 95.13% respectively, highlighting how each enhances feature representation at different levels.
(3) The ALGA module, by adaptively combining local and global attention, enhances feature expression. Removing ALGA drops accuracy to 93.17%, underscoring its importance in integrating feature information. The DMA module, which dynamically adjusts the importance of features, increases classification accuracy; its removal lowers accuracy to 94.65%, highlighting its essential role in processing multimodal data. Simultaneous removal of ALGA and DMA reduces accuracy to 92.79%, illustrating the critical synergy of these modules in enhancing model performance.
(4) The complete model performs best across all experiments, achieving an accuracy of 97.94%. This confirms the effectiveness of combining various modules to enhance ground-based cloud classification. Through multilevel and multimodal feature extraction and fusion, ALGA-DenseNet significantly enhances performance.

In summary, each module plays a crucial role, and their synergistic interaction further enhances the overall model performance. These results validate the effectiveness of the proposed network structure in ground-based cloud classification.

An ablation study was conducted to validate the impact of the Color Jitter augmentation strategy on model performance. The results reveal a significant performance disparity: the model trained with color jitter achieved an accuracy of 97.94%, a substantial improvement of 6.85% over the 91.13% accuracy of the model trained without it. This quantitative leap suggests that color jitter is critical for forcing the model to learn robust, generalizable features intrinsic to cloud morphology, rather than overfitting to superficial chromatic cues like specific lighting or time-of-day color tones.

**Fig 12:**
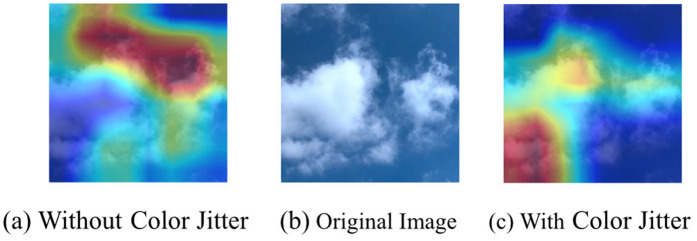
Class Activation Maps.

To offer insight into how Color Jitter affects model behavior, we conducted a qualitative analysis using Class Activation Maps (CAMs), which visualize the regions of an image most influential to the model’s classification decision. The results, presented in Fig 12, suggest a shift in the model’s learning focus. As shown in Fig 12a, the baseline model without Color Jitter exhibits diffuse attention, with activation often spreading into irrelevant background areas, suggesting a reliance on broad color or textural cues. In contrast, Fig 12c shows that our final model learns to concentrate its attention more effectively on the primary cloud structures. While not perfectly delineating the edges, the high-activation regions are more closely aligned with the main body of the cloud. This comparison provides qualitative evidence that our strategy encourages the model to prioritize more structurally relevant features, such as general shape and texture, over superficial and unreliable color cues. This shift helps explain both the significant boost in accuracy and the model’s improved focus.

### 4.3 Comparative experiments

To verify the effectiveness of the proposed ALGA-DenseNet architecture, it was benchmarked against well-known neural network models using the same ground-based cloud dataset. The models included ResNet50, AlexNet, VGG16, MobileNetV2, GooLeNet, and the ALGA-DenseNet introduced in this paper. Classification accuracy results for each model are as follows:

The results in Table 4 demonstrate that the proposed ALGA-DenseNet outperforms all other models, achieving a classification accuracy of 97.94%. GooLeNet ranks second with 93.45%, followed by MobileNetV2 at 92.27%. ResNet50, AlexNet, and VGG16 achieved accuracies of 90.37%, 89.65%, and 88.74%, respectively.

**Table 4:** Single Model Comparative Experiment.

Model	Accuracy
Densenet	85.87%
ResNet50	90.37%
AlexNet	89.65%
VGG16	88.74%
MobileNetV2	92.27%
GooLeNet	93.45%
**ALGA-DenseNet**	**97.94%**

These results highlight the superior performance of ALGA-DenseNet, demonstrating its effectiveness in accurately classifying ground-based cloud data. The significant increase in ALGA-DenseNet’s accuracy is due to its advanced feature extraction and fusion capabilities, combining adaptive local and global attention mechanisms with dynamic multimodal attention. These mechanisms enable the model to effectively capture and integrate relevant features from multiple modalities, resulting in more accurate predictions.

Moreover, the use of depthwise separable convolutions reduces the number of model parameters, significantly lowering computational complexity and enhancing efficiency while maintaining high performance. Models using traditional connections achieved an accuracy of 94.58%, while the element-wise multiplication approach in ALGA-DenseNet reached 97.94%. The significant improvement in accuracy demonstrates the higher efficacy of element-wise multiplication for feature fusion, enhancing the model’s ability to represent complex data. Overall, comparative experiments confirm that ALGA-DenseNet not only improves classification accuracy but also illustrates the potential of using sophisticated attention mechanisms and multimodal fusion techniques in complex classification tasks within deep learning models.

**Fig 13:**
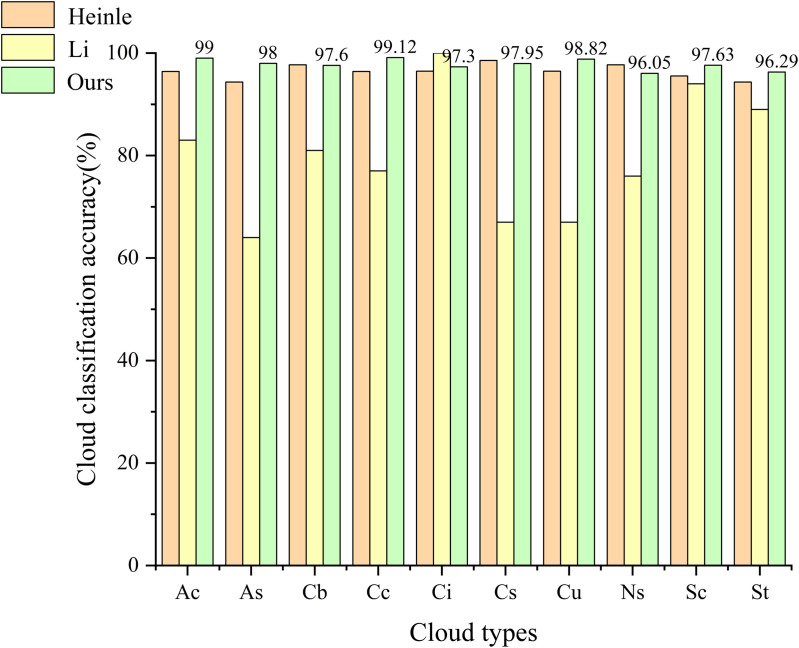
Comparative Graphs of Different Methods.

To better assess model performance, the results were compared with those of several other methods, as shown in Table 5. Our method demonstrates considerable progress, and it is important to note that all competing methods were reimplemented and evaluated by us under identical conditions to ensure a fair comparison. Furthermore, to verify the model’s broad applicability, we also trained it on the CCSN dataset. The CCSN [19] dataset, the Cirrus-Cumulus-Stratus-Nimbus Cloud Dataset, comprises 2,543 annotated ground-based cloud images captured from a ground-based platform across 11 categories: altocumulus, stratocumulus, nimbostratus, cumulus, cirrus, cirrocumulus, cumulonimbus, altostratus, contrails, cirrostratus, and stratus. As shown in Fig 13, we compared accuracy across ten categories, finding slightly lower performance in the Cs and Ns categories compared to Heinle’s method. Observations of images from the Cs and Ns categories revealed larger cloud cover areas and densities, where Heinle’s feature extraction performed better. Our method achieved higher accuracy in other categories than both Heinle and Li [31], and on average, still outperformed other methods.

**Table 5:** Comparative Results of Different Methods.

Method	Accuracy
Heinle [25]	93.66%
Liu [26]	79.8%
Zhi [30]	88.1%
**Ours**	**97.94%**

As shown in Table 6, the model demonstrated strong classification capabilities on the CCSN dataset, achieving an accuracy of 97.25%, and performed excellently across individual categories and overall, indicating good generalizability and adaptability.

**Table 6:** Different Dataset Results.

Model	Accuracy	Recall	F1-Score
GCD	97.94%	0.9764	0.9779
CCSN	97.25%	0.9728	0.9729

## 5 Conclusion

The ALGA-DenseNet model proposed in this paper has achieved significant success in ground-based cloud classification, demonstrating exceptional performance and innovation. It achieved a classification accuracy of 97.94% on the GCD dataset and 97.25% on the CCSN dataset. Compared to other classic models, ALGA-DenseNet excels in both classification accuracy and generalization.

By incorporating ALGA mechanisms, the model adaptively enhances crucial texture and color details in cloud imagery while suppressing irrelevant background noise, significantly improving feature representation and classification performance. The ALGA module preserves essential color information during feature fusion, aiding in the differentiation of various cloud types.

Integrating depthwise separable and mixed convolutions enhances the model’s ability to capture multi-scale features, which is crucial for recognizing the varied shapes and textures of clouds. Utilizing convolutional kernels of various sizes allows the model to capture texture features at different scales effectively, from detail to the whole. Moreover, these convolution operations significantly reduce the number of parameters and computational complexity.

By integrating the ViT and DMA, the model captures long-range dependencies, providing a comprehensive understanding of cloud shapes and structures. DMA dynamically adjusts attention weights, facilitating adaptive focus on crucial features, thereby enhancing the model’s accuracy and robustness.

Comprehensive ablation studies confirm the essential role of each module in enhancing model performance, particularly the synergistic effect of the ALGA and DMA modules, which not only maintain but also improve performance while reducing parameter count.

However, we acknowledge certain limitations that present avenues for future work. A key consideration is the inherent trade-off of our color augmentation strategy. While Color Jitter proved highly effective at enhancing robustness against illumination variance, we recognize that aggressive color transformations risk obscuring subtle, physically meaningful chromatic cues related to cloud microphysics. For the GCD dataset, however, our ablation study results and the improved feature localization in the Class Activation Maps strongly suggest that the benefits of mitigating lighting inconsistencies far outweighed the potential loss of this microphysical information. Further challenges for real-world deployment, such as for UAV safety, stem from the datasets used. The current study is validated on datasets that do not include complex meteorological conditions such as fog, haze, or transitional cloud states. Additionally, the dataset’s single “Mixed” label for complex scenes and its fixed ground-based perspective limit the model to scene-level recognition from a specific viewpoint, rather than enabling multi-label classification or view-invariant analysis. The GCD dataset’s taxonomy is based on visual similarity for machine vision rather than distinct meteorological formation mechanisms, a grouping which may mask critical distinctions. Therefore, future research should focus on a dual objective: collecting more diverse, real-world data to develop models robust to challenging conditions, and simultaneously exploring or curating datasets with a more physically meaningful taxonomy. Training advanced models like ALGA-DenseNet on such improved benchmarks is a crucial next step toward building truly reliable systems for safety-critical applications.

In summary, the ALGA-DenseNet model proposed in this paper demonstrates excellent performance and high computational efficiency in the 7-category ground-based cloud classification task, offering significant practical value. Future research could further optimize the model structure and explore its application under diverse meteorological conditions. A particularly promising direction is the deployment of this model on edge devices, such as those integrated into UAVs, to provide real-time, on-board cloud analysis. This would enhance autonomous navigation and mission planning for UAVs operating in complex weather environments. Furthermore, incorporating additional meteorological parameters, such as air pollution indices that affect visibility, could further improve the model’s generalization and classification accuracy in real-world scenarios.
